# Utilizing a family-based economic strengthening intervention to improve mental health wellbeing among female adolescent orphans in Uganda

**DOI:** 10.1186/s13034-019-0273-4

**Published:** 2019-03-11

**Authors:** Apollo Kivumbi, William Byansi, Fred M. Ssewamala, Nabunya Proscovia, Christopher Damulira, Phionah Namatovu

**Affiliations:** 1 International Center for Child Health and Development, P.O. Box 1988, Circular Rd, Masaka, Uganda; 2 New York University Silver School of Social Work, New York, USA; 3 Brown School, Washington University in St. Louis, St. Louis, USA

## Abstract

**Background:**

It is estimated that almost 20% of the world’s adolescents have experienced or are experiencing a mental health problem. Several factors have been associated with the onset of adolescent mental health disorders, including poverty, child abuse and violence, particularly among adolescent girls. This paper examines the effect of participating in a family-based economic strengthening intervention on the mental health well-being of female adolescent orphans impacted by HIV/AIDS in rural Uganda.

**Methods:**

Data utilized in this study was from the Bridges to the Future Study (2011–2016), an economic empowerment intervention aimed at improving health outcomes of orphaned children. Adolescents were randomly assigned to either the control condition receiving bolstered standard of care services for orphaned adolescents; or one of two treatment conditions receiving bolstered standard of care as well as an economic empowerment intervention comprising of a child development account, a mentorship program and workshops on financial management and microenterprise development. Data was collected at baseline, 12- and 24-months post intervention initiation. Multilinear regression analyses were conducted to examine the impact of an economic empowerment intervention on mental health functioning of female participants over time. Mental health functioning was measured by: (1) the Child Depression Inventory; (2) Beck Hopelessness Scale; and (3) Tennessee Self Concept Scale.

**Results:**

Analysis results show an improvement in mental health functioning over time among female participants receiving the intervention compared to their control counterparts. Specifically, compared to participants in the control condition, participants receiving the intervention reported a reduction in depressive symptoms from baseline to 12-months follow-up (b = − 1.262, 95% CI − 2.476, − 0.047), and an additional 0.645-point reduction between baseline and 24-months follow-up (b = − 1.907, 95% CI − 3.192, − 0.622). Participants receiving the intervention reported significant improvement in their reported self-concept from baseline to 24 months follow-up (b = 3.503 (95% CI 1.469, 5.538) compared to participants in the control condition.

**Conclusions:**

Empowerment of young girls, either in the form of peer mentorship and/or economic strengthening seems to significantly improve the overall mental health functioning of adolescent girls impacted by HIV and AIDS in low-income settings.

## Introduction

Worldwide, an estimated 10–20% of adolescents and youth between 14 and 24 years old struggle with mental health issues [1]. Among these, depression is the third leading disability among adolescents between the ages of 15–19 years [2]. Available data from Sub-Saharan Africa (SSA) indicates that 1 in 7 children and adolescents (between 0 and 16 years) had a mental health difficulty [3]. Disease and poverty have intrinsically exacerbated mental health problems [3]. For example, available data from cross sectional surveys indicate that 21% of adolescents in Uganda suffer from mental disorders like depression [4].

Among orphaned and vulnerable children (OVCs), it has been widely documented that poor mental health functioning is associated with several negative health and social outcomes which persists even into adulthood [5–8]. Specifically, orphaned adolescents, especially those orphaned due to HIV/AIDS, experience higher levels of mental health problems and exhibit higher levels of psychological distress compared to those orphaned due to other causes [9–11]. Parental illness and death increase economic burden, loss of social support, and despair about the future [12–15]. Orphaned adolescents may not directly express their worries and anxieties, leading to feelings of anger, resentment as well as a sense of alienation and desperation [16]. Such emotions can result in risk taking behaviors and withdrawal [16]. As such, orphaned adolescents need extra social support to help them cope with these challenges as they transition into adulthood.

In addition, studies have documented that female adolescents are more likely to exhibit severe emotional and behavioral distress compared to adolescent boys. For example, Rescorla et al. [17] found that female adolescents reported higher levels of overall emotional distress and more depressive symptoms than did male adolescents. Other studies have documented that female orphans exhibit higher levels of psychological distress, social isolation, loss of education, and risky behaviors compared to boys [10, 18]. Therefore, it is critical to examine programs and interventions that address mental health risks among female adolescents made vulnerable by HIV and AIDS.

Household and community-level poverty exacerbate the mental health challenges of orphaned female adolescents. In the face of this reality, researchers have been testing asset-based interventions that utilize Child Development Accounts (CDAs) to address some of the mental health challenges associated with orphanhood [19, 20]. These efforts utilize asset theory, which suggests that there is a positive link between economic assets and psychological wellbeing [21, 22]. Specifically, owning assets, such as savings, educational opportunities, economic opportunities, including microenterprise activities, has important social, economic and psychological benefits. In addition, owning assets positively impacts the individuals’ behaviors, attitudes, future orientation and hopes for the future [23]. Indeed, these studies have found that economic opportunities results in positive mental health outcomes among orphaned adolescents in SSA, including reduced depressive symptoms and hopelessness [20, 24] and improving future orientation and self-concept [19]. Other positive social and economic outcomes relate to savings, improved educational outcomes, and reduced risk taking behaviors [25, 26].

Building upon asset theory and previous findings, this current study examines both the short-and long-term impact of a family-based economic strengthening intervention on the mental health wellbeing of female adolescents orphaned by HIV/AIDS. We argue that female adolescents participating in an economic empowerment intervention that combines matched savings, peer mentorship, financial management trainings and income generating activities will be more likely to report higher levels of self-concept, lower depression levels and more hopefulness about the future compared to their counterparts in the control condition. Unlike previous findings, this study utilized data from a relatively larger sample of adolescents, and outcomes are tracked over a longer period of time. The results from this study expand on similar findings from other SSA countries and provide practical implications that can inform efforts aimed at developing comprehensive programs for female orphans.

## Methods

### Study setting

The current study used data from the *Bridges to the Future Study* (2011–2016), a randomized experimental study examining a family-based economic empowerment intervention for children orphaned by HIV/AIDS in rural Uganda. The *Bridges* study was conducted in 4 geopolitical districts of Masaka, Rakai, Kalungu & Lwengo in southern Uganda—a region heavily affected by HIV and AIDS.

#### Study population

**Fig. 1:**
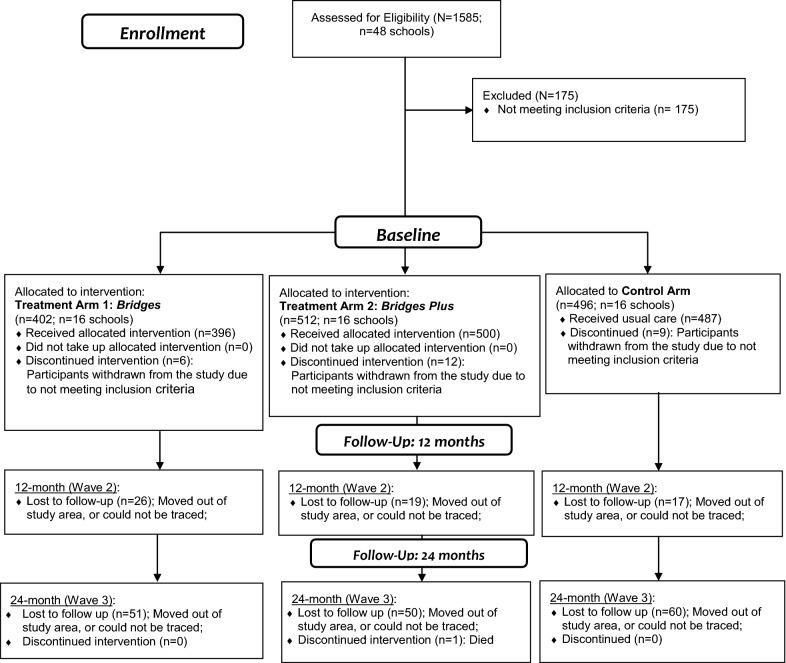
Consort flow diagram

Randomization was done by an independent research fellow who was not part of the study. A total of 789 female orphaned adolescents between the ages of 10–16 years were recruited from 48 geographically separated day public primary schools with comparable performance at national standardized examination, and similar socioeconomic characteristics of the student’s body. Participants were eligible to participate in the study if they: (1) identified as orphans, having lost one or both parents to HIV/AIDS, (2) were living within a family, not an institution, and (3) enrolled in grades 5 or 6 of a government aided primary school at the time of study recruitment (Fig. 1).

#### The *Bridges* intervention

Detailed information on study design and implementation is provided elsewhere [10, 27]. In brief, participants in the Bridges study were randomly assigned at the school level to three groups. The control arm (n = 273 girls) received bolstered usual care services for orphaned children, consisting of scholastic materials such as notebooks, text books, school lunches and school uniforms. The two treatment arms i.e. Bridges arm (n = 228 girls) and Bridges plus arm (n = 288 girls) received usual care services mentioned above, plus three intervention components: (1) an economic empowerment intervention consisting of Child Development Accounts (CDAs) with matched savings. Participants’ savings were matched at a level of 1:1 for Bridges arm and 2:1 for the Bridges plus arm. All savings were intended to pay for post-primary education and or microenterprise development, which study facilitators relayed to participants; (2) workshops on financial management and training in income generating activities (IGA); and (3) mentorship with peer mentors throughout the intervention period. For the purposes of this paper, the two treatment groups were combined into one group.

Data utilized in this paper was collected at baseline, 12- and 24-months post intervention initiation. Data was collected via interviewer administered surveys, each lasting approximately 90 min.

#### Measures

Adolescent mental health was assessed using three measures: depression, hopelessness and self-concept. All assessments were conducted by research assistants trained in Good clinical practices and CITI certified. The measures were administered in the local language.

Depression was measured using the Child Depression Inventory [28]. The 27-item tool assesses self-reported depressive symptoms over the past 2 weeks. Sample items, coded as 0, 1, 2 include: “*I hate myself, I do not like myself, I like myself”*. The Cronbach’s alpha was 0.68, indicting moderate reliability. Items in the inverse direction were reverse coded to create summated scores, with higher scores indicating higher levels of depressive symptoms.

Hopelessness was measured using the Beck Hopelessness Scale [29]. The 20-item tool measures hopelessness and pessimistic attitudes towards the future. The reliability test yielded a Cronbach’s alpha of 0.66. Response categories were ‘true” (coded as 0) or “false” (coded as 1). Sample items included: “*I can’t imagine what my life will be like in 10* *years’ time”.* Summary scores were calculated, with high scores indicating higher levels of hopelessness.

Participants’ self-concept was measured using the Tennessee Self Concept Scale [30]. The 20-item tool measures children’s perception of identity, self-satisfaction and other behaviors. Responses were rated on a 5-likert point scale, with 1 = always false & 5 = always true. The scale yielded an acceptable Cronbach’s alpha of 0.74. Sample items include: “*I like the way I look, and I have a happy family.*” Summary scores were calculated with high scores indicating high levels of self-concept.

The primary independent variable was participation in the economic empowerment intervention. This variable was coded as “1” for participants in the treatment condition and “0” for participants in the control condition. The two treatment arms were combined into one group. Further, the analysis controlled for participants’ socio-demographic characteristics including age, orphan hood status (maternal, paternal, and double orphan), primary caregiver (biological/surviving parent, grandparent(s), or other relative), household composition (number of adults and number of children residing in the household), adolescent physical health (poor to fair, good or excellent), and availability of savings and family relationship levels.

#### Data analysis

We ran both simple and multiple linear regression models for each of the outcome variables (i.e. depression, hopelessness and self-concept) controlling for participants’ sociodemographic characteristics. Specifically, we conducted t-tests and Chi square to determine the difference in means of demographic characteristics. In addition, we conducted multiple linear regression analysis to compute average changes in outcome measures between baseline (wave I), 12-months (wave II) and 24-months follow-up (wave III). All analyses were conducted in STATA 14.1. For this analysis we combined both treatment arms and compared them to the control arm because there was no statistically significant difference between the treatment arms.

## Results

From Table 1, the total number of girls in the study was 789 with 273 (36.6%) in the control arm and 516 (63.4%) in the combined treatment arms. Among the participants, 80.6% (*n *=220) and 78.5% (*n *= 405) in control and treatment arms respectively considered themselves to be in good to excellent physical health. Among the participants in the treatment arm, most reported their caretaker as a living biological parent (40.3%, *n *=208) while for those in control, 38.5% (*n *= 38.5) are taken care of by grandparents. Respondents who had lost both parents were 21.3% (*n *= 58) and 17.2% (*n *= 89) in control and treatment study arms respectively. Individual savings were reported to be at 21.3% (*n *= 58) and 23.8% (*n *= 123) among respondents in control and treatment arms respectively. The average age of respondents in the control group was 12.58 years, whereas that average age of respondent in the combined treatment arms was 12.34 years (*p*-value 0.007). There was no significant difference (*p*-value 0.455) in family cohesion mean scores between control (23.52) and treatment (23.33) groups. The average number of adults (above 18 years) per household was 6.52 and 6.24 for control and treatment respondents respectively, while children per household (less than 18 years excluding the participant) were 3.32 for control and 3.15 for treatment respondents on average.

**Table 1:** Socio-economic characteristics at baseline (*N *= *789*)

Characteristic	Control (n = 273)	Treatment (n = 516)	t-value or χ^2^
Age, *mean* [*range* 10–16]	12.582	12.341	2.7246**
Family cohesion, *mean* [*range* 7,30]	23.52	23.233	0.7475
Household composition			
Total no. of people in HH			
*Mean* [*range* 2,19]			
Adults	6.52	6.238	1.3691
*T *= *Mean* [*range* 0,15]			
Children	3.322	3.151	1.0788
Orphan hood status (n, %)			3.2010
Paternal	153 (56.0)	322 (62.4)	
Maternal	62 (22.7)	105 (20.4)	
Double	58 (21.3)	89 (17.2)	
Personal savings (n, %)			0.6784
Yes	58 (21.3)	123 (23.8)	
No	215 (78.7)	393 (76.2)	
Primary caregiver (n, %)			2.3199
Biological parent	95 (34.8)	208 (40.3)	
Grand Parent	105 (38.5)	184 (35.7)	
Other relatives	73 (26.7)	124 (24.0)	
Physical health (n, %)			0.4772
Poor to fair	53 (19.4)	111 (21.5)	
Good to excellent	220 (80.6)	405 (78.5)	

** p ≤ 0.01

From Table 2, respondents demonstrated an average reduced risk of depression with the intervention in both the unadjusted − 1.35 (95% *CI* − 2.55, − 0.15, p ≤ 0.05) and adjusted models − 1.262 (95% *CI* − 2.476, − 0.047, p ≤ 0.05) between waves I and II. The same average risk reduction in depression was observed between waves I and III in both unadjusted − 2.004 (*95% CI* − 3.247, 0.761, p ≤ 0.01) and adjusted models − 1.907 (*95% CI* − 3.192, − 0.622, p ≤ 0.01). However, respondents did not show significant change in risk reduction in depression between wave II and wave III. In terms of self-concept, there was an average significant increase in the self-concept of respondents in the treatment arm compared to those in the control arm between waves I and II in both the unadjusted and adjusted models; 3.714 (*95% CI* 1.53, 5.898, p ≤ 0.01) and 3.503 (*95% CI* 1.469, 5.538, p ≤ 0.001) respectively. The observed average improvement in self-concept in the unadjusted model between wave 1 and III, 2.631 (*95% CI* 0.056, 5.206) was not observed in the adjusted model, 2.272 (*95% CI* − 0.261, 4.804). No risk difference in hopelessness was observed among participants at any point in time.

**Table 2:** Treatment effects on mental well-being of female participants between different waves of data collection

Mental health measure	Unadjusted Risk difference	Adjusted Risk difference
Depression		
Wave I–wave II	*−* *1.351 (−* *2.553, −* 0.15)*	*−* *1.262 (−* *2.476, −* 0.0474)*
Wave II–wave III	− 0.533 (− 1.341, 0.276)	− 0.508 (− 1.378, 0.363)
Wave I–wave III	*−* *2.004 (−* 3.247, 0.761)**	*−* *1.907 (−* *3.192, −* 0.622)**
Self-concept		
Wave I–wave II	3.714 (1.53, 5.898)***	3.503 (1.469, 5.538)***
Wave II–wave III	− 1.576 (− 4.147, 0.995)	− 1.795 (− 4.459,0.869)
Wave I–wave III	2.631 (0.056, 5.206)*	2.272 (− 0.261,4.804)
Hopelessness		
Wave I–wave II	− 0.532 (− 1.139, 0.076)	− 0.565 (− 1.143,0.012)
Wave II–wave III	0.374 (− 0.17, 0.917)	0.467 (− 0.085, 1.019)
Wave I–wave III	− 0.215 (− 0.952, 0.522)	− 0.127 (− 0.809,0.556)

* p ≤ 0.05; ** p ≤ 0.01; *** p ≤ 0.001: all models controlled for participants’ socio-economic characteristics

## Discussion

While several interventions have been utilized to improve the mental health functioning for female adolescents, this study focused on utilizing an economic empowerment approach to reduce depression levels and hopelessness and improve the self-concept of school-going female adolescent orphans made vulnerable by HIV/AIDS. Female adolescents who received the economic empowerment intervention in the form of a CDA combined with peer mentorship, IGA training and financial education, reported lower levels of depression and higher self-concept over time compared to their counter parts in the control condition. The changes in participants’ mental health functioning could be attributed to the fact that participating in empowerment programs provided a sense of hope, positive views of the future and economic resources needed to deal with daily financial challenges that cause stress among female adolescents. Findings have documented that lacking familial support, poverty [3, 19, 31, 32], and being affected by HIV and AIDS [14, 15] negatively impact mental health among children made affected by HIV and AIDS. Additionally, access to economic resources led to an increase in female adolescents’ decision-making abilities over a number of life events, which is quality that results in better mental health functioning, as has been documented by other studies [24]. Therefore, the provision of economic means to deal with the stressors caused by poverty and death of parents can potentially lower depression levels. Furthermore, participating in economic empowerment brings families together to discuss strategies for saving and making investment plans. Consistently, research indicates that parents and/or caregivers that provide emotional and informational support to their children can help lower their depression and enhance their self-concept [9, 33].

The major limitation of this study is the reliance on self-report which is susceptible to social desirability bias. In addition, given than this was a combination intervention, it is difficult to singularly assess the effects of each intervention component on the wellbeing of female adolescents.

### Implications

Findings from this study point to two major implications. First, economic empowerment interventions have the potential to improve the overall mental health functioning of female adolescent orphans living in low-income settings. Second, multiple component interventions, such those that combine economic empowerment, peer mentorship, financial education and IGA training can have multiple positive outcomes among orphaned adolescents.
